# Loneliness and Depression: The Mediating Effect of Perceived Social Strain in Older Adults with Diabetes

**DOI:** 10.1093/geroni/igaf122.2783

**Published:** 2025-12-31

**Authors:** Emma Cho, George Demiris

**Affiliations:** University of Pennsylvania, Philadelphia, Pennsylvania, United States; University of Pennsylvania, Philadelphia, Pennsylvania, United States

## Abstract

Loneliness is a known risk factor for social strain which refers to the capability of certain social relationships to act as a source of stress. Social strain has been shown to negatively affect mental health outcomes such as depression in older adults. Older adults with diabetes are at greater risk for loneliness. However, limited research has examined there is a lack the complex relationship among loneliness, social strain, and depression in this population. The purpose of this study was to examine the relationship between loneliness and depression in older adults with diabetes and to explore the mediating role of social strain in this association. This cross-sectional study was developed as a secondary data analysis using data from the National Social Life, Health, and Aging Project in the USA. The sample consisted of 379 older adults aged 50 to 88 years. Loneliness, social strain, and depression were assessed using a multi-item survey questionnaire. Bivariate analysis and macro PROCESS in SPSS were used to analyze the data. Mediation analysis revealed a positive, direct effect of loneliness on depression (path c’: β = 1.61, p <.001), and a positive, indirect effect of loneliness on depression through perceived social strain (β = 0.20, p <.001). These findings suggest that the relationship between loneliness and depression is partially mediated by social strain. Interventions that target both loneliness and the negative perceptions or interactions associated with social strain could help reduce depression in this population.

